# Supplementary material for: Downregulation of S6 Kinase and Hedgehog–Gli1 by Inhibition of Fatty Acid Synthase in AML with FLT3-ITD Mutation
Source: Int J Mol Sci. 2025 Jun 14;26(12):5721. doi: 10.3390/ijms26125721 (PMC12193305; doi:10.3390/ijms26125721)
Supplement: Supplementary file 1 [file ijms-26-05721-s001.zip › ijms-3642900-supplementary.pdf]

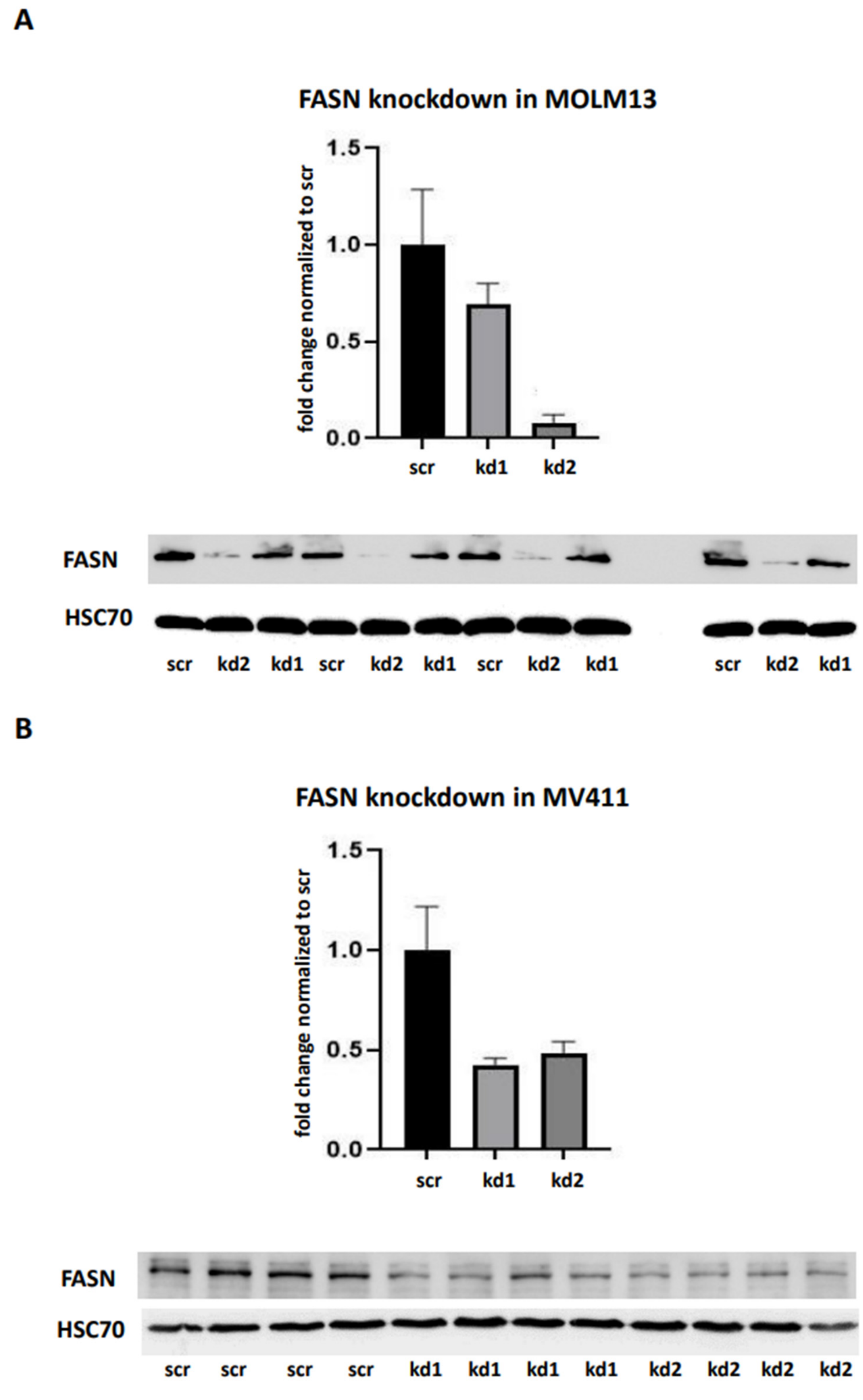

Supplementary Figure S1: Verification of the shRNA-mediated knockdown of endogenous FASN in MOLM13 and MV411 cells.

The MOLM13 and MV411 cell lines were transduced with FASN shRNA (vector 1, referred to as kd1, or vector 2, referred to as kd2), or with a non-targeting vector (scr) as a control for baseline FASN expression. Protein lysates were analyzed by Western blot to assess FASN expression in technical quadruplicates for both cell lines, with HSC70 used as a loading control for FASN quantification. FASN expression levels in kd1 and kd2 were normalized to those in the scr control. Reduction of FASN expression was observed for both kd1 and kd2 in MOLM13 and in MV411. Densitometric quantification of the phospho-protein/protein ratios was performed after normalization to scr control, and the results are presented as mean values with standard deviations.

A

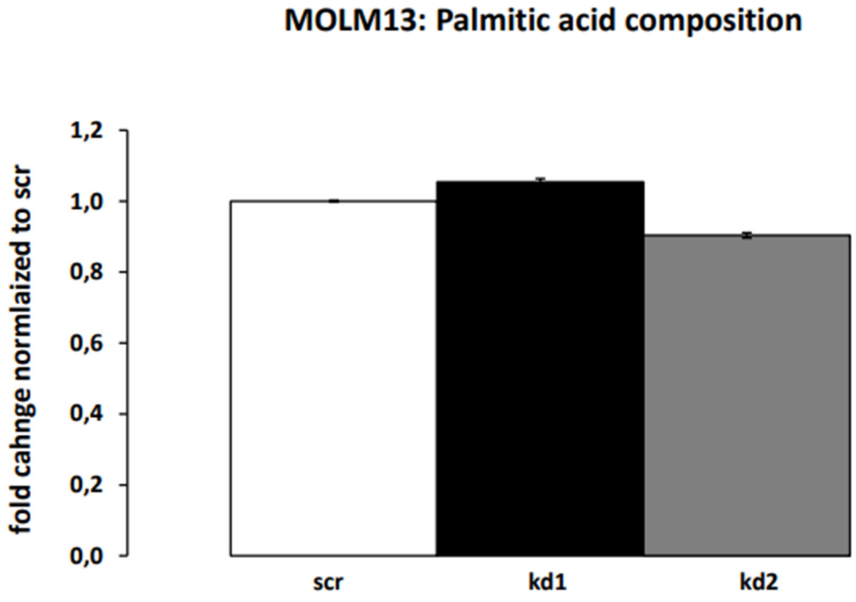

B

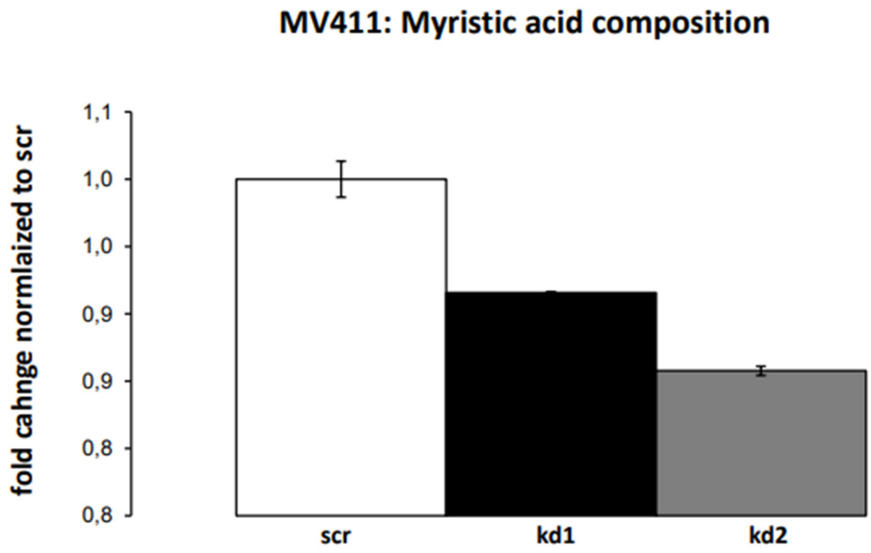

Supplementary Figure S2: Lipidomic analysis in MOLM13 and MV411 cells upon FASN knockdown. The concentrations of myristic and palmitic acids were measured relative to the total fatty acid content and normalized to scr. All samples were analyzed in technical duplicates. In MOLM13 (upper diagram), FASN kd2 led to a 10% reduction in palmitic acid levels. In MV411 (lower diagram), kd1 and kd2 resulted in reductions of myristic acid levels by 8% and 14%, respectively.

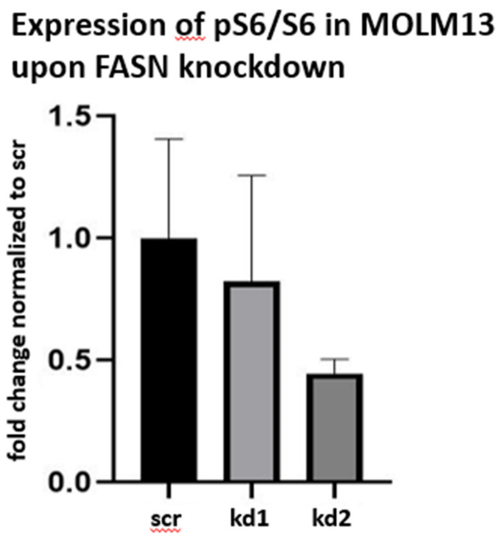

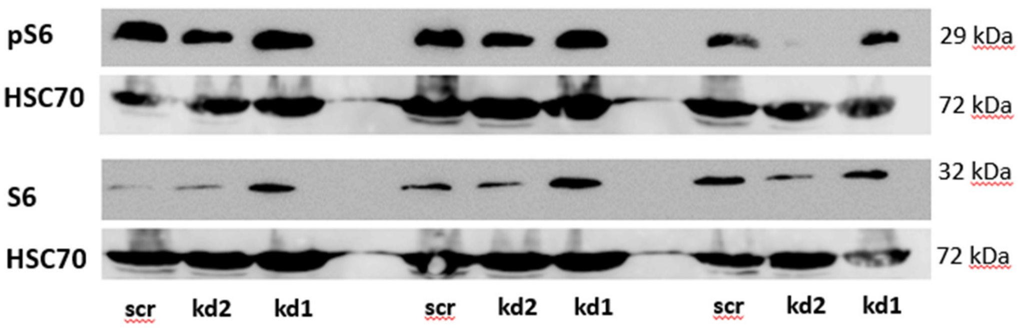

Supplementary Figure S3: Analysis of pS6 expression in FASN knockdown MOLM13 cells by Western blot. Protein lysates from MOLM13 cells were analyzed to evaluate the expression of pS6 and total S6 (S6) in technical triplicates, with HSC70 used as a normalization control. The expression levels of pS6 and S6 in kd1 and kd2 were normalized to scr. Stable knockdown of FASN (kd2) resulted in a numeric decrease in pS6 levels compared to scr. Densitometric quantification of the phospho-protein/protein ratios was conducted after normalization to scr, with the results presented as mean values and standard deviations.

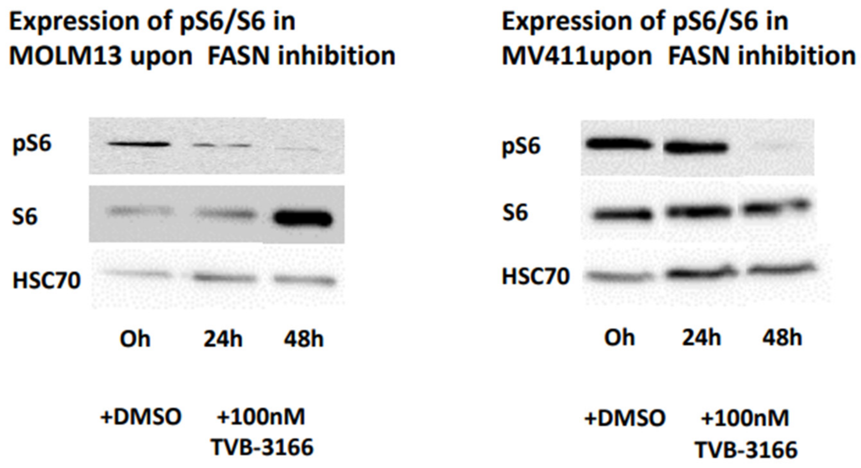

Supplementary Figure S4: Verification of pS6 downregulation in MOLM13 and MV411 cells upon treatment with the FASN inhibitor TVB-3166 by Western blot. Protein lysates from MOLM13 and MV411 cells were analyzed to assess the expression of pS6, and total S6 (S6) with HSC70 serving as a normalization control. In TVB-3166-treated cells, pS6/S6 expression was compared to dimethylsulfoxid(DMSO)-treated controls. In both MOLM13 and MV411 cells, treatment with 100nM TVB-3166 resulted in reduced pS6 levels compared to DMSO treatment. Densitometric quantification of the phospho-protein/protein ratios was performed after normalization to scr, and results are presented as mean values ± standard deviations.

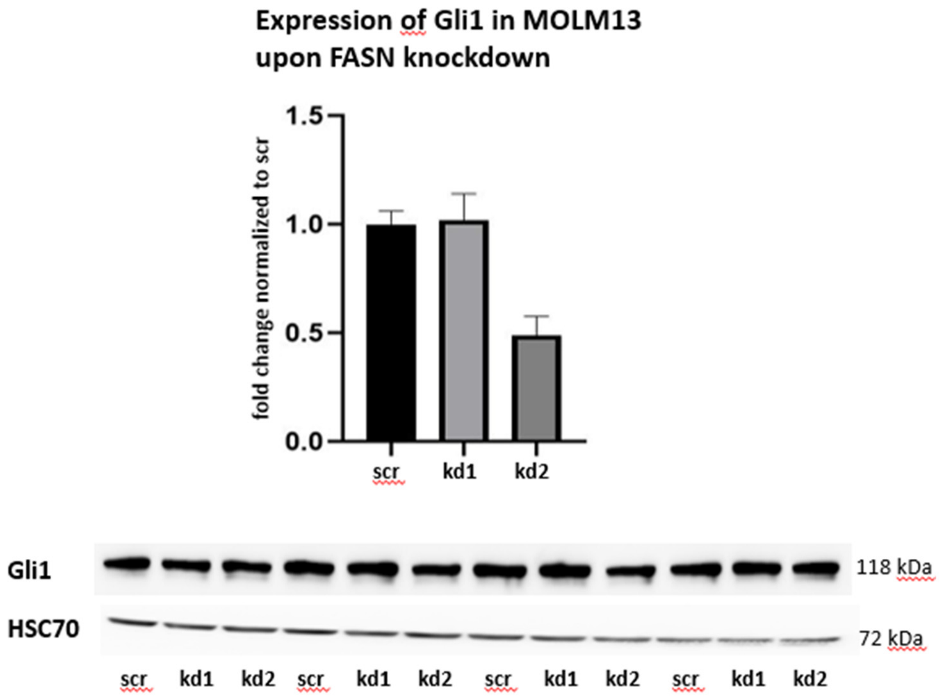

Supplementary Figure S5: Verification of the downregulation of Gli1 in FASN knockdown MOLM13 cells by Western blot. Protein lysates from MOLM13 cells were analyzed to assess the expression of Gli1 with HSC70 serving as a normalization control. In kd2 MOLM13 cells, stable FASN knockdown led to decreased Gli1 expression compared to scr. Densitometric quantification of the phospho-protein/protein ratios was performed after normalization to scr, and results are presented as mean values ± standard deviations.

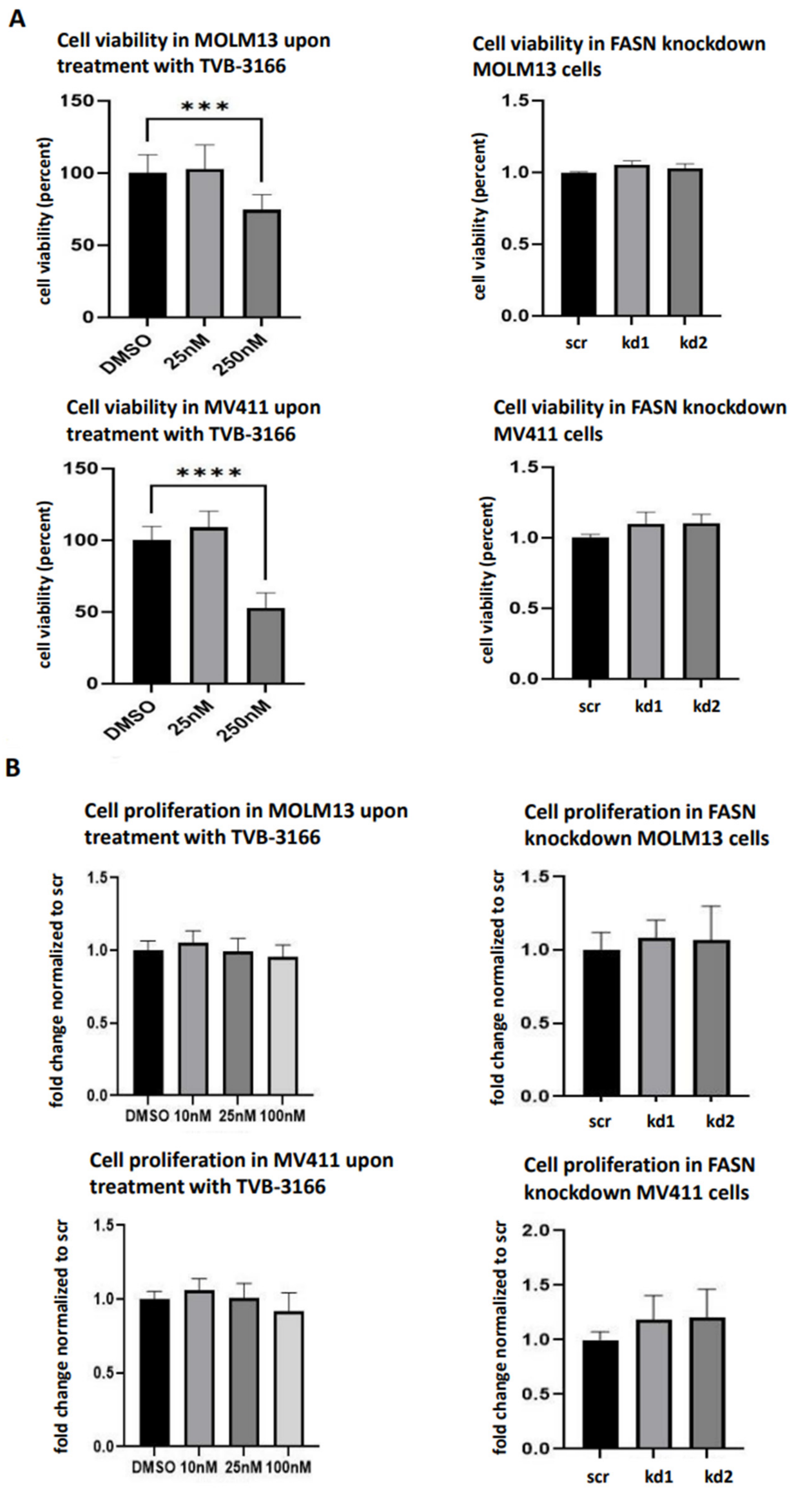

Supplementary Figure S6: Cell viability (A) and cell proliferation (B) in MOLM13 and MV411 cells upon inhibition of FASN by TVB-3166 as well as by FASN knockdown.
Cell viability was assessed on day 5 following inhibitor treatment using the Vi-CELL™ XR, employing a trypan blue exclusion assay. Statistical significance is denoted by *** for $p \leq 0.001$, and by **** for $p \leq 0.0001$. **A**: Treatment with 250nM TVB-3166 resulted in a significant reduction in cell viability in both cell lines compared to DMSO, whereas 25nM TVB-3166 did not significantly affect cell viability. No changes in viability were observed in either MOLM13 or MV411 kd1 and kd2 cell lines with stable FASN knockdown compared to scr control. **B**: No significant changes in cell proliferation were observed in either MOLM13 or MV411 cells following FASN knockdown or treatment with TVB-3166 at doses ranging from 10nM to 100nM.
